# Long-term evolution of antibiotic tolerance in *Pseudomonas aeruginosa* lung infections

**DOI:** 10.1093/evlett/qrad034

**Published:** 2023-09-20

**Authors:** Melanie Ghoul, Sandra B Andersen, Rasmus L Marvig, Helle K Johansen, Lars Jelsbak, Søren Molin, Gabriel Perron, Ashleigh S Griffin

**Affiliations:** Department of Biology, University of Oxford, Oxford, United Kingdom; Center for Evolutionary Hologenomics, Globe Institute, University of Copenhagen, Copenhagen, Denmark; Center for Genomic Medicine, Rigshospitalet, Copenhagen, Denmark; Department of Clinical Microbiology, Afsnit 9301, Rigshospitalet, Copenhagen Ø, Denmark; Department of Clinical Medicine, Faculty of Health and Medical Sciences, University of Copenhagen, Copenhagen, Denmark; Department of Biotechnology and Biomedicine, Technical University of Denmark, Lyngby, Denmark; Department of Clinical Medicine, Faculty of Health and Medical Sciences, University of Copenhagen, Copenhagen, Denmark; Center for Environmental Sciences and the Humanities, Bard College, Annandale-On-Hudson, NY, United States; Center for Genomics and Systems Biology, New York University, New York, NY, United States; Department of Biology, University of Oxford, Oxford, United Kingdom

**Keywords:** adaptation, evolutionary medicine, microbial evolutionary genomics

## Abstract

Pathogenic bacteria respond to antibiotic pressure with the evolution of resistance but survival can also depend on their ability to tolerate antibiotic treatment, known as tolerance. While a variety of resistance mechanisms and underlying genetics are well characterized in vitro and in vivo, an understanding of the evolution of tolerance, and how it interacts with resistance in situ is lacking. We assayed for tolerance and resistance in isolates of *Pseudomonas aeruginosa* from chronic cystic fibrosis lung infections spanning up to 40 years of evolution, with 3 clinically relevant antibiotics: meropenem, ciprofloxacin, and tobramycin. We present evidence that tolerance is under positive selection in the lung and that it can act as an evolutionary stepping stone to resistance. However, by examining evolutionary patterns across multiple patients in different clone types, a key result is that the potential for an association between the evolution of resistance and tolerance is not inevitable, and difficult to predict.

## Introduction

The ability of bacterial cells to survive antibiotic treatment can arise from genetically determined resistance and phenotypic tolerance. Experimental and epidemiological research showing how this ability to survive evolves in response to treatment, focusses on the evolution of resistance (Santi et al., 2021; Bartell et al., 2020; Lopatkin et al., 2021). In contrast, the extent to which tolerance is under positive selection in infections is poorly understood (Balaban et al., 2019; Bigger, 1944; Hobby et al., 1942). Furthermore, predictions about how the evolution of resistance and tolerance are associated are untested due to a lack of data characterizing changes in tolerance and resistance over time in situ (Fisher et al., 2017). One possibility is that phenotypic tolerance can facilitate the evolution of genetic resistance through enhanced survival and, therefore, the opportunity for resistance mutations to arise (Santi et al., 2021; Mulcahy et al., 2010). To test this prediction, we characterized long-term evolution of resistance and tolerance in *Pseudomonas aeruginosa* from chronic lung infections of individuals with cystic fibrosis (CF).

Bacterial tolerance describes the ability of cells to adopt a phenotype that survive prolonged exposure to external stressors, such as a bactericidal drug concentration (Balaban et al., 2019; Bigger, 1944; Hobby et al., 1942). Persistence is specifically defined as a subset of these tolerant cells in an isogenic population that can survive the antibiotic (Balaban et al., 2019). A level of consensus has been reached on how to define and quantify tolerance and persistence (Balaban et al., 2019). A specific gene responsible for persistence has been found only for *Escherichia coli*, while a range of regulatory and metabolic genes and mechanisms that contribute to or correlate with tolerance have been identified in *P. aeruginosa*, as well as other pathogenic species (Balaban et al., 2019; De Groote et al., 2009; Drescher et al., 2019; Fisher et al., 2017; Santi et al., 2021; Schumacher et al., 2015; Viducic et al., 2017). Tolerance is often attributed to dormant or slowly dividing cells (Balaban et al., 2004; Gollan et al., 2019) including those in biofilms (Olsen, 2015), although dormancy has been shown to not always be necessary (Orman & Brynildsen, 2013). Regardless of whether the mechanisms of tolerance are multiple or yet unknown (Balaban et al., 2019), progress can be made on the key question of whether it is under positive selection in infections.

What are the current theories about how selection for tolerance operates? One hypothesis is that tolerance is favored as a bet-hedging strategy (Bakkeren et al., 2020) when antibiotic concentration is high but exposure infrequent (Gardner et al., 2007; Kussell & Leibler, 2005; Kussell et al., 2005; Van den Bergh et al., 2016b). Tolerance may also evolve in response to intermediate concentrations and facilitate subsequent evolution of resistance (Bakkeren et al., 2019; Levin-Reisman, Gefen, & Balaban, 2017; Levin-Reisman, Ronin, et al., 2017; Windels et al., 2019): A population of bacterial cells may be more likely to evolve resistance if it can survive for a sufficient number of generations to allow beneficial mutations to arise. There is rarely more than 1% of an in vitro population in a tolerant state at any given time, but this is sufficient to re-colonize once antibiotic pressure has been released (Balaban et al., 2019). If tolerance is adaptive, we expect to see its frequency increase under strong selection from antibiotics. Could such an increase contribute to the failure to treat chronic infections? Antibiotic tolerance in *P. aeruginosa* pathogens was observed repeatedly and early in infection (Bartell et al., 2020; Mulcahy et al., 2010) and was found to be selected prior to resistance in vitro (Cohen et al., 2013; Michiels et al., 2016; Mulcahy et al., 2010; Nguyen et al., 2011; Santi et al., 2021). This suggests that it is a heritable trait, but do we observe similar patterns of its emergence in situ?

Here, we test how antibiotic response strategies of *P. aeruginosa* evolve during CF infection, by measuring and comparing changes in tolerance and resistance to three antibiotics used in the clinic. This infection system is ideal for detecting selection on tolerance because long-term sampling has occurred from lineages of transmissible strains, infecting for 40 years (Bartell et al., 2020). We use a collection of isolates from individuals with CF consisting of two transmissible clone types, and we track the evolution of resistance and tolerance in 107 clinical isolates sampled longitudinally from 27 individuals over a period of four decades (Supplementary Table S1). These comparisons allow us to detect evidence of a role for tolerance in the transition from acute to chronic infection. The three antibiotics we used to challenge the isolates in this collection have different modes of action: ciprofloxacin, a fluoroquinolone that interferes with DNA replication; meropenem, a beta-lactam that inhibits cell-wall synthesis; and tobramycin, an aminoglycoside that inhibits protein synthesis. We further investigate if the bacterial survival strategy is general or specific to the type of antibiotic, and whether there is an interaction between resistance and tolerance.

## Results

### Selection on tolerance in infection

#### Patterns of tolerance evolution in transmissible lineages

**Figure 1.**
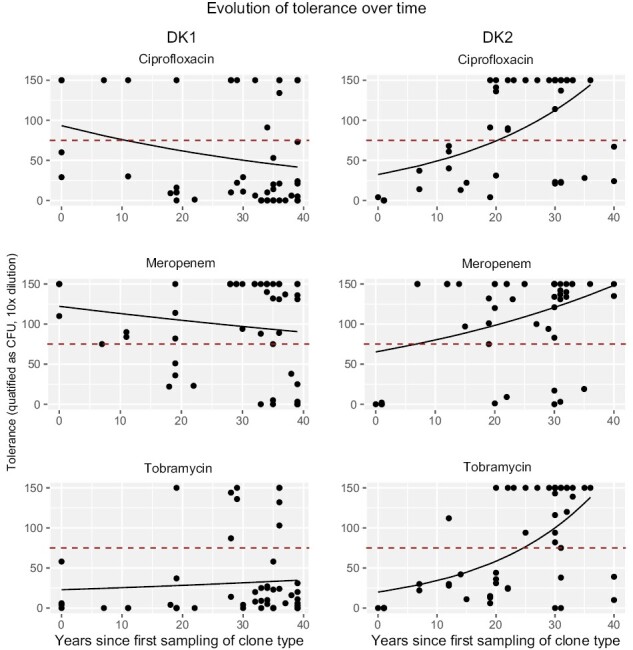
Graphs show number of tolerant cells per isolate, measured as colony-forming units (CFUs), over time for the two different clone types and three different antibiotics, and the GLM fit using time to predict change in CFU. The dashed red line marks the cut-off of 75 CFU to classify isolates as either low or high tolerance. On the left, DK1, and on the right, DK2. Tolerance was measured as CFU counts at 10 times dilution of culture.

We found strong evidence for positive selection on tolerant cell formation in both transmissible strains tested (Figure 1), but patterns did not support tolerance as a general response to antibiotic treatment in infection. In the transmissible strain DK2, we found positive selection in all three antibiotics treatments. More specifically, we found a significant increase in tolerant cell counts per isolate over time in response to all three experimental antibiotic treatments (GLMs, *p* < .05; Supplementary Table S2, Figure 1). We first observed high tolerance (>75 colony-forming units [CFUs] per isolate) after 19 years of sampling across patients in response to ciprofloxacin, after seven years in response to meropenem, and after 12 years for tobramycin.

For the DK1 lineage, tolerant cell formation increases significantly with length of infection in our tobramycin treatment, with high tolerance first observed after 19 years. In contrast, while high tolerance is observed in both early and late DK1 isolates in response to ciprofloxacin and meropenem, there is a significant decrease in tolerance over time (GLMs, *p* < .05; Supplementary Table S2, Figure 1).

### Resistance is under positive selection in infection

**Figure 2.**
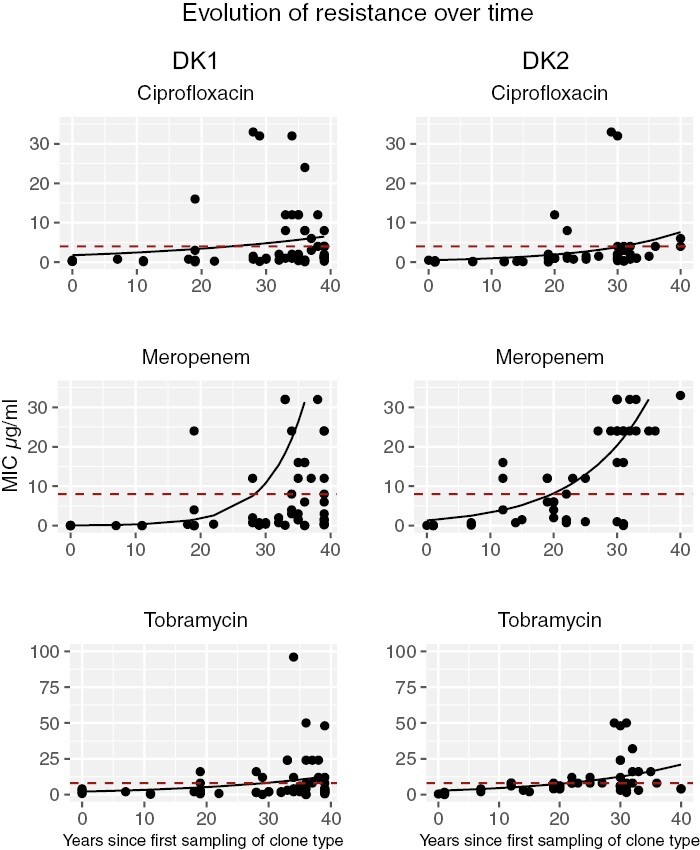
Graphs show change in resistance (minimum inhibitory concentration [MIC]) over time in the transmissible clone types DK1 (left side) and DK2 (right side), to three different antibiotics (GLM fits). Dashed red lines indicate clinical cut-off for classification of resistance (MIC of 4 for ciprofloxacin, and 8 for meropenem and tobramycin). Eight DK1 isolates with an MIC > 33 are not shown for meropenem).

For the transmissible clone types, there is a significant increase in antibiotic resistance, measured as minimum inhibitory concentration (MIC), to all three antibiotics, for both clone types (DK1: 57 isolates sampled between 1973 and 2012; and DK2: 50 isolates sampled between 1972 and 2007; GLMs, *p* < .05; Supplementary Table S3, Figure 2). However, it takes over a decade for clinically resistant mutants (see Methods) to become detectable: resistance to ciprofloxacin is first observed after 20 years of infection and after 12 years for meropenem and tobramycin. It is important to note that no data are available on when treatment with each antibiotic type was initiated in patients, which influences resistance onset.

### Evolution of resistance and tolerance within transmissible lineages

#### Tolerance can act as a stepping stone to resistance

**Figure 3.**
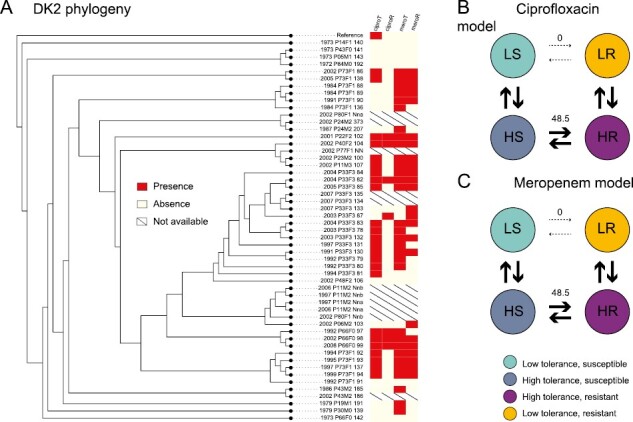
(A) The phylogeny of clone type DK2 isolates with the tolerance (T) and resistance (R) phenotypes to ciprofloxacin (cipro, first two columns) and meropenem (mero, last two columns; red: presence, white: absence, strike-through: phenotype unknown). Branch lengths are not drawn to scale. Panels (B) and (C) show the modeled transition rates between phenotypic states, indicated by the stroke and number on arrows. We find that the dependent model fits the data best as resistance is unlikely to evolve before tolerance, from the wild-type susceptible and low tolerance phenotype. This is also reflected in (A), as resistance is only found in the absence of high tolerance once for ciprofloxacin and twice for meropenem. The dependent model for tobramycin was not significantly different from the null hypothesis of independent evolution of tolerance and resistance and is not shown.

**Figure 4.**
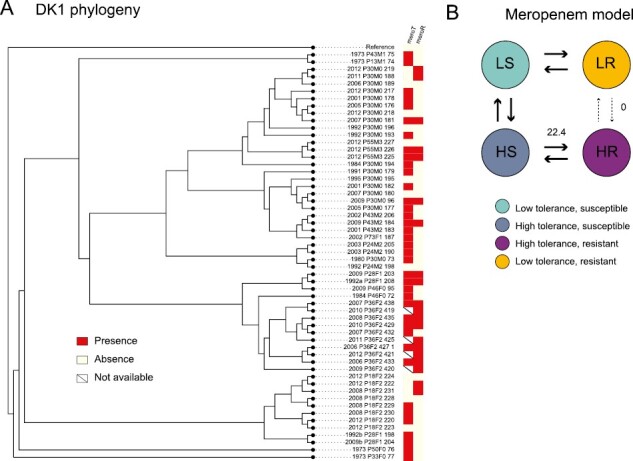
(A) The phylogeny of clone type DK1 isolates with the tolerance (T) and resistance (R) phenotypes to meropenem (mero; red: presence, white: absence, strike-through: phenotype unknown). Branch lengths are not drawn to scale. Panel (B) shows the modeled transition rates between phenotypic states, indicated by the stroke and number on arrows. We find that the dependent model fits the data best as the high tolerance resistant phenotype is unlikely to evolve from the low tolerance resistant state. The dependent models for ciprofloxacin and tobramycin were not significantly different from the null hypothesis of independent evolution of tolerance and resistance and are not shown.

Given that resistance and tolerance are under positive selection, we next test how these traits might be associated with transmissible lineages. We find that tolerance can act as a stepping stone to resistance. The patterns we observe are distinct for the different experimental antibiotic treatments and clone types. For DK2, the evolution of resistance depends on the evolution of tolerance, for both ciprofloxacin (likelihood ratio: 4.84; *p* = .028) and meropenem (likelihood ratio: 3.62; *p* = .057; Figure 3). For DK1 for meropenem, the first isolates are tolerant, and we see that while tolerance can act as a stepping stone to resistance, the reverse is significantly less likely to occur (*p* = .033; Figure 4). For tobramycin, and ciprofloxacin for DK1, no significant patterns were observed.

### Tolerant phenotypes correlate across antibiotics in one transmissible lineage

**Figure 5.**
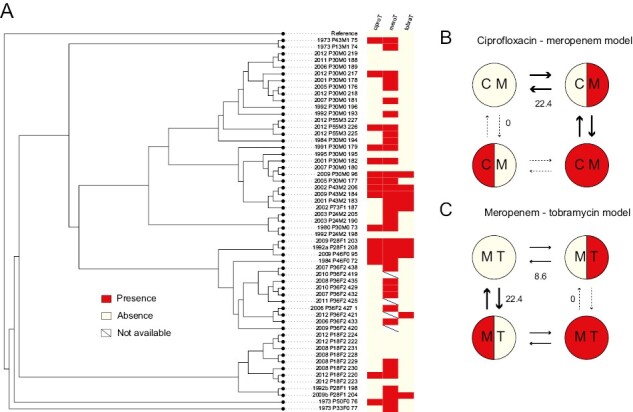
(A) The phylogeny of clone type DK1 isolates with the tolerance phenotypes (red: tolerance, white: low tolerance, strike-through: phenotype unknown) to the three antibiotics mapped. (B) For tolerance to ciprofloxacin and meropenem, the dependent model fits the data best as tolerance to ciprofloxacin is unlikely to evolve before tolerance to meropenem. This is also reflected in (A), as all isolates that are tolerant to ciprofloxacin also show tolerance to meropenem. Numbers and size of arrows indicate the modeled rate of transition, given the simplest model that assumes that all rates are equal. (C = ciprofloxacin, m = meropenem). (C) Similarly, for meropenem and tobramycin, the dependent model fits the data best as tolerance to tobramycin is unlikely to evolve before tolerance to meropenem. The dependent model for ciprofloxacin and tobramycin was not significantly different from the null hypothesis of independent evolution and is not shown.

After exploring the evolution of tolerance to an individual antibiotic, we test if there are patterns in response across the three antibiotics. For DK1, we see a correlation, as tolerance to ciprofloxacin evolves after tolerance to meropenem (likelihood ratio: 18.36; *p* = .0001, Figure 5A). Furthermore, tolerance to meropenem is more likely to evolve before tolerance to tobramycin and act as a stepping stone to tolerance to both (likelihood ratio: 5.76; *p* = .016; Figure 5B). No significant correlations were found for DK2 (see all test results in SOM).

We observe the independent evolution of high tolerance to all three antibiotics seven times, and the same for resistance (Supplementary Figure S1A and B). For five of these, there is an overlap in that the same isolates show resistance and tolerance to all antibiotics.

### Isolates with low population density have higher levels of resistance and tolerance

**Figure 6.**
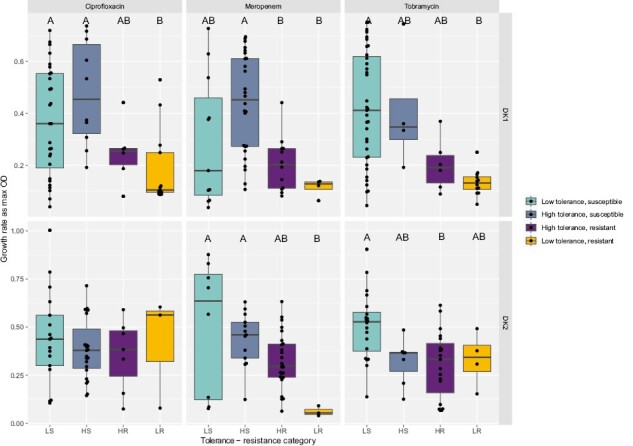
Population density measured as max OD for tolerance-resistance categories. Boxplots show median max OD ±25 percentiles and values as dots. Grouping by –two-way ANOVA Tukey HSD test, *p* < .05 denoted by letters A–C, groups are significantly different if they do not share a letter (Supplementary Table S4). LS = low tolerance, susceptible; LR = low tolerance, resistant; HS = high tolerance, susceptible; HR = high tolerance, resistant.

**Figure 7.**
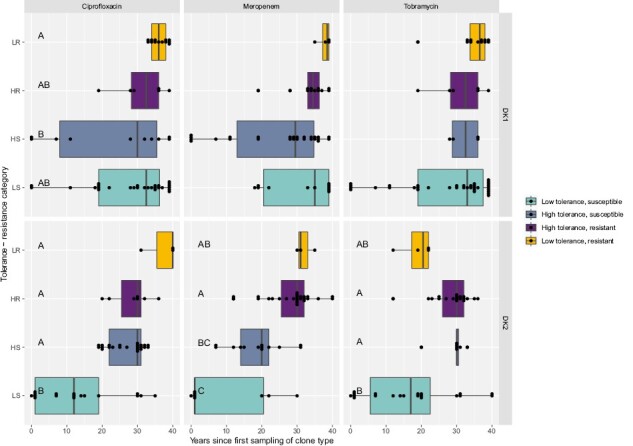
Isolate sampling time for tolerance-resistance categories. Boxplots show median sampling time ±25 percentiles and values as dots. Grouping by –two-way ANOVA Tukey HSD test, *p* < .05 denoted by letters A–C (Supplementary Table S5). LS = low tolerance, susceptible; LR = low tolerance, resistant; HS = high tolerance, susceptible; HR = high tolerance, resistant.

We find that maximum bacterial density after 24 hr of growth is negatively correlated with both tolerance and resistance. This indicates that resistant and tolerant isolates either grow slower or have an extended lag and/or stationary phase. Resistant isolates that are either high or low tolerance generally have significantly lower population densities (purple and yellow boxes in Figure 6; Supplementary Table S4). There was, however, also a significant decrease in maximum density with infection time regardless of the resistant or tolerant phenotypes (linear regression; DK1: *R*^2^_adj_ = .50, *p* < .001; DK2: *R*^2^_adj_ = .21, *p* < .001). So, the negative correlation between resistance and high tolerance with low density could be confounded by the fact that resistant isolates tend to be sampled late in infection (Figure 7). Analyzing the data including both sampling time and population density to test for their effect on the evolution of resistance and tolerance, shows that lower population density in itself is associated with an evolved antibiotic response (except for tolerance to meropenem treatment in DK2; Supplementary Tables S2 and S3).

## Discussion

We investigated the evolution of antibiotic tolerance and resistance in two transmissible *P. aeruginosa* clone types over an estimated 1.3 million generations (Andersen et al., 2018). We found evidence of positive selection on both tolerance and resistance to three clinically relevant antibiotics: meropenem, ciprofloxacin, and tobramycin. Both strategies take time to evolve; high tolerance evolves de novo after 7–19 years and clinical resistance after 12–20 years. One striking pattern is that tolerance can act as a stepping stone to resistance. This result is consistent with experimental evolution studies of resistance and tolerance in *P. aeruginosa* (Santi et al., 2021) and *E. coli* (Levin-Reisman, Gefen, & Balaban, 2017; Levin-Reisman, Ronin, et al., 2017). However, the pattern was not consistent across drugs and clone types. Despite intensive antibiotic treatment over more than 35 years, tolerance to two out of three antibiotics did not increase over time for the DK1 clone type. This emphasizes the need for caution in making assumptions about how bacterial cells should respond to antibiotic treatment. Other mechanisms, such as biofilm production or limitations to diffusion of antibiotics play a role in the establishment of bacterial infection, despite antibiotic treatment early in infection (Hall & Mah, 2017; Olsen, 2015; Rossi et al., 2020).

The collection of isolates we work with is unique in the extent of sampling in time and across patients, enabling us to correctly treat patients rather than co-infecting cells as independent data points in our analyses (Jelsbak et al., 2007; Markussen et al., 2014; Marvig et al., 2013; Rau et al., 2012; Yang et al., 2011). However, it should be noted that from most time points only one isolate is available. While infections are typically dominated by a single or two clonal lineages, populations can be heterogeneous (Markussen et al., 2014; Winstanley et al., 2016; Workentine et al., 2013), and we may miss rare phenotypes, and only detect ones that later reach a certain frequency. These effects will have reduced our ability to detect patterns of evolution.

We find that patients are typically colonized by antibiotic susceptible and non-tolerant phenotypes, and that strategies to evade the effect of antibiotics are, unsurprisingly, favored in the lung (Figures 1–3). The pattern of how these traits evolve over time differs dramatically, however, between the two transmissible lineages we examined. One of the lineages, DK2, shows a significant increase in tolerance over time in response to all three antibiotics, while DK1 only shows increased tolerance under tobramycin treatments and a significant decrease in tolerance under meropenem and ciprofloxacin treatments. These differences may reflect phylogenetic topology: an early isolate of DK2 acquired several mutations and founded a successful lineage, which we sampled in subsequent generations (Marvig et al., 2013, Supplementary Figure S1B). In contrast, the DK1 phylogeny has several distinct subclades that evolve independently (Supplementary Figure S1A). Some clades maintain “wild-type-like” production of virulence factors after more than 30 years of infection (e.g., the siderophore pyoverdine [Andersen et al., 2015] and protease [Markussen et al., 2014]). The ancestral state of DK1 is high tolerance to meropenem treatment, and high tolerance to ciprofloxacin has evolved nine times independently (Supplementary Figure S1A) since 1973 (Figure 1). Overall, after 40 years of infection, some isolates still exhibit low tolerance or susceptibility to one antibiotic; however, very few are susceptible and low tolerance to *all* antibiotics (DK1: seven isolates sampled from 1992 to 2012; DK2: one isolate sampled in 1992), perhaps reflecting differences in drug treatment (Jorth et al., 2015; Markussen et al., 2014).

An outstanding question is how specific tolerance is to antibiotic type. In one clone type, we found a significant overlap in tolerance between ciprofloxacin and meropenem, and meropenem and tobramycin. Tolerance to one drug may act as a stepping stone to tolerance to another. A recent study (Santi et al., 2021) shows the correlation of the tolerant phenotype between ciprofloxacin and tobramycin. The comparison with our findings perhaps further emphasizes the importance of clone type-specific effects. In short, significant caution is required in extrapolating the results from a study on one strain to antibiotic resistance and tolerance in general and emphasizes the value of examining the complexity of bacterial traits in situ as well as examining these traits in the lab.

Working with clinical isolates also offers the opportunity to examine and observe response to treatment that may not be anticipated in advance. We found that some isolates from early infection, with a high tolerance phenotype, exhibit a “revival” phenotype to meropenem and/or ciprofloxacin. This is defined as showing no growth in undiluted culture, but growth appearing again following dilution (Supplementary Figure S2; light red circles in Supplementary Figure S1). This phenotype indicates the presence of tolerant cells that are only detected when the antibiotic is removed from their environment. This “revival” phenotype is only observed in the early isolates, and we speculate that this could be a potential intermediary state to high tolerance. All other isolates classified as tolerant had high CFU counts from both undiluted and 10-fold diluted cultures. The correlation between meropenem and ciprofloxacin could arise from the antibiotic killing mode and bacterial resistance mechanisms: *P. aeruginosa* cells can tolerate ciprofloxacin exposure by elongating without dividing, allowing cells to survive longer in the antibiotic such that when it is removed they can divide and grow (Brazas et al., 2007; Goormaghtigh & Van Melderen, 2019; Kohanski et al., 2010). Meropenem can be enzymatically degraded by beta-lactamase activity (Ciofu et al., 2000). This detoxifies the environment from meropenem at a faster pace, allowing cells to revive faster (Medaney et al., 2016). In contrast, tobramycin disrupts bacterial membranes, killing cells upon exposure to high concentrations (Bulitta et al., 2015), such that cells either persist or die with no revival intermediary. This may also explain why we observe fewer high-tolerant isolates in the presence of tobramycin treatment (Figure 6).

Our finding that maximum density is negatively correlated with both tolerance and resistance is consistent with tolerant cells either growing more slowly or having an extended lag and/or stationary phase. Both of these traits have been observed to contribute to tolerance (Fridman et al., 2014; Lewis, 2007; Pontes & Groisman, 2019; Stepanyan et al., 2015; Van den Bergh et al., 2017) and are a common phenotype in chronic infections (Rossi et al., 2020). Tolerant cells are thought to be relatively dormant with reduced metabolic rate, found deep within biofilm layers (Olsen, 2015). It has also been shown that resistance often comes at a metabolic cost that may lower the growth rate (Martinez & Rojo, 2011; Wong et al., 2012). Additionally, because later stages of infection, in our study, are characterized by isolates that achieve smaller populations densities in vitro but higher tolerance counts, this indicates that the fraction of tolerant cells in the population will be proportionally higher in these compared to isolates with a higher maximum density. However, it is important to note that slow growth is a common phenomenon in *P. aeruginosa* isolates from late-stage infection, and this may be influenced by factors other than antibiotic response strategies (Rossi et al., 2020).

Resolving the genetics of antibiotic resistance in *P. aeruginosa* is notoriously challenging, as resistance generally is caused by SNPs, and there are few plasmids (Botelho et al., 2019). Despite the intensive study of the isolate collection, clear genetic patterns have not been found (Rossi et al., 2020). For tolerance, the genetic mechanisms are even less characterized (Balaban et al., 2019). It is a complex, polygenic trait, that may be under selection by variables other than antibiotics, such as oxidative stress and host immune evasion, and also be affected by selection on other phenotypes such as growth rate. It is necessary to focus on the phenotype rather than the genotype of tolerance, which has not been well characterized. With this study, we hope to contribute a better understanding of how the evolution of tolerance and resistance are associated; the effect of traits such as growth rate; and associations with different types of antibiotics. This is important because the discovery of new antibiotics has stalled, and our best approach is to use the already available drugs in novel ways—by reconsidering doses and combinations (Hansen et al., 2020; Imamovic & Sommer, 2013). To do so, we must continue to explore how bacteria respond and adapt to drugs in situ.

In conclusion, our results indicate that tolerance and resistance phenotypes show limited evolution before a decade post-infection of patients from which we obtained samples. In the 1970s, when these samples were collected, antibiotic therapy was also less aggressive and so the selective pressure is likely higher in contemporary patients. Tolerance is observed earlier in infection than resistance, when it could be used as a stepping stone to resistance and thereby contribute to the development of recalcitrant infections. Resistance seems to be the primary strategy adopted by bacterial cells for surviving antibiotic treatment in late-stage, chronic infections. Overall, our study highlights the importance of resisting generalization when drawing conclusions from studies on single clones and/or single antibiotics to better understand tolerance. We cannot assume every infection follows the same course but that each is influenced by pathogen genetics, antibiotic treatment and the host environment. Critical to achieve this is the development of methods for easy detection of tolerance, which is not routinely screened for. These results could have implications for early intervention treatment strategies to prevent the evolution of tolerance and ultimately resistance (Van der Bergh et al., 2016a), e.g., using lower doses of antibiotics and in specific combinations, allowing us to prolong the use of antibiotics for treating infections (Hansen et al., 2020; Imamovic & Sommer, 2013).

## Materials and methods

### Isolate collection

The collection of isolates is from Danish cystic fibrosis individuals sampled at Rigshospitalet, Copenhagen and consists of samples from 27 individuals infected with two clone types that were transmitted within the clinic (57 DK1 isolates from 13 individuals sampled between 1973 and 2012, and 50 DK2 isolates from 18 individuals, sampled between 1972 and 2007; (Jelsbak et al., 2007; Markussen et al., 2014; Marvig et al., 2013; Rau et al., 2012; Yang et al., 2011); see Supplementary Table S1). The maximum likelihood DK2 phylogeny, based on the sequence alignment from Marvig et al. (2013) and done in RAxML version 8.2.11, suggests that a successful line evolved early, which was transmitted between all subsequent individuals, whereas the DK1 phylogeny (Andersen et al., 2018) is less well resolved with more distinct subclades (Supplementary Figure S1A and B). Length of infection for DK1 and DK2 represents time since the first isolate of the clone type was recorded in 1972 or 1973, respectively. We excluded eight DK2 isolates from the phylogeny as high-quality sequence data was not available, and only used data from these in the time series analyses. Due to the presence of hyper-mutator isolates in the collection, some branch lengths in the phylogenies were much longer than others (Andersen et al., 2018; Marvig et al., 2013). To make the figures easier to view, we transformed phylogenies in FigTree v1.4.2 (https://github.com/rambaut/figtree/releases) using the *transform branches* setting “proportional.”

### Quantifying antibiotic resistance

To prepare cultures, we grew freezer stock overnight in 6 ml liquid LB media at 37 °C with shaking and then standardized cultures to an optical density 0.5 McFarland standard. We then assayed antibiotic resistance in these cultures by swabbing bacterial suspensions onto Mueller-Hinton agar plates with three ETEST plastic strips placed on top in accordance with the manufacturer’s instructions (Liofilchem, Italy). ETEST strips have a predefined gradient of antibiotic concentration (Marley et al., 1995), which allowed us to measure MIC values. The concentrations measured were the following: ciprofloxacin: 0.002–32 µg/ml, meropenem: 0.002–32 µg/ml, and tobramycin: 0.016–256 µg/ml. We scored the isolates after 24 hr as sensitive, intermediate or resistant according to the species-specific MIC clinical breaking points user guide available from Rosco Diagnostica (http://pishrotashkhis.com/wp-content/uploads/2017/07/Neo-SENSITAB-CLSI-EUCAST-Potency.pdf), and as defined by Clinical and Laboratory Standards Institute (CLSI.org). The MICs were measured on agar plates using strips, which has been shown to be comparable to values obtained in liquid culture (Baker et al., 1991) and therefore supporting our antibiotic tolerance assays done in liquid media.

### Quantifying antibiotic tolerance

We cultured isolates overnight in 6 ml liquid LB media and incubated them at 37 °C with shaking. We then standardized the culture to an optical density (OD) of *A*_600_ = 0.1. From this, we inoculated 2 µl into 200 µl of LB media in 96-well plates and incubated them at 37 °C for 24 hr. As a proxy for growth rate and population size, we measured OD and calculated the mean of the replicates after 24 hr of growth in media. After the 24 hr, we added antibiotic to each well at a concentration of 100 µg/ml (meropenem, tobramycin, or ciprofloxacin). We incubated the cultures for 24 hr at 37 °C and subsequently plated them out on LB agar plates (see below). Isolates were replicated eight times for each antibiotic. In the meropenem treatment, four isolates that have MIC values above 100 µg/ml were removed from the persister analysis.

Following incubation with antibiotics, we diluted the *P. aeruginosa* cultures 10 and 100-fold using 0.9% NaCl solution. Undiluted, 10 and 100-fold dilutions were plated onto LB agar plates in 5 µl drops to count the viable CFUs. Maximum number of CFUs counted was set to 150. For each isolate, we calculated the mean CFU and standard deviation around the mean from the replicates at each dilution. As we cap the CFU count at 150, we do not calculate tolerance as a fraction of the starting population density (OD). However, as population size decrease with time, while persistence increases (see below), this results in a conservative estimate of tolerance.

We did not create kill curve assays for the full collection of isolates, as is required to distinguish between different types of tolerant cells per the recent classifications defined by Balaban et al. (2019). Initial experiments on a subset of the clinical isolates showed that they behave differently to PA01, and classical kill curves were not observed. Rather, we observed no CFU counts for the first 15 hr post-antibiotic treatment at 0, 10, and 100-fold dilutions. For this reason, we chose to only get end point reads of CFU counts at 24 hr, which represents the plateau of the kill curve.

### Longitudinal changes in antibiotic tolerance and resistance during infection

We analyzed the evolution of tolerance and resistance for the transmissible clone types DK1 and DK2, using generalized linear models (GLMs), to test the relationship between tolerance (as CFU counts) and resistance (as MIC values), with length of infection and initial density after 24 hr growth.

### Classifying resistance and tolerance

The isolates’ MIC for each antibiotic range from 0.008 to 32 µg/ml for ciprofloxacin; 0.006–48 µg/ml for meropenem (with the exception of four isolates which have been removed from the persister analysis), and 0.064–32 µg/ml for tobramycin. We classified the isolates based on the clinically relevant cut-offs (4 µg/ml for ciprofloxacin, and 8 µg/ml for meropenem and tobramycin as defined by Clinical and Laboratory Standards Institute, CLSI.org), and with a conservative approach, grouped the intermediately resistant isolates with the susceptible in subsequent analyses. Resistance phenotypes of the isolates to the three antibiotics are mapped to the phylogeny in Supplementary Figure S1A and B. The antibiotic concentration we used for the tolerance assays was significantly above the MIC, i.e., *ca*. between 2 and 17,000 times higher. It has been found that as long as a concentration above the MIC is used, the difference between the MIC and the applied amount does not correlate with the level of tolerance observed as found in pilot experiments using the lab strain, PA01. Any growth (observed as CFU counts) from antibiotic treated cultures are considered to be a result of tolerance, defined here as the ability to survive exposure to the antibiotic at values above the clinical MIC cut off.

We chose to use CFU counts from the 10-fold dilution for all antibiotics to optimize the number of isolates with countable CFUs (>1 and <150), and facilitate comparison across antibiotics and isolates (Supplementary Table S1). The majority of isolates did not exhibit a clear 10-fold dilution in CFU (i.e., they had similar numbers of CFU at 10- and 100-fold dilutions, or >150 CFU at 10-fold and 0 at 100-fold). Residual antibiotics in the 10-fold dilution of the liquid culture may have inhibited growth of tolerant cells, resulting in more CFUs at 10-fold dilution than on plates of undiluted culture. This was only observed in 11/321 cases and we classify these as a “revival” category (Supplementary Figure S2). In only 2/321 cultures (both in the tobramycin treatment) would a 100-fold dilution have resulted in a difference in classification from low to high tolerance (see below); an insufficient number to impact on our overall results.

We set 75 CFUs as a cut-off, where isolates with counts above this are categorized as “high tolerance,” and those below categorized as “low tolerance.” Tolerance phenotypes of the isolates to the three antibiotics are mapped to the phylogeny in Supplementary Figure S1A and B. There was a bell-shaped relationship between mean CFU count and the value of the standard deviation around the mean, which reflects that isolates with mean CFU counts between 25 and 100 have a large variation between replicates, rather than an intermediate number of CFUs (Supplementary Figure S3). The midpoint count of 75 CFUs is at, or above the peak of the standard deviation. Isolates with < 75 CFUs will have more replicates with no CFUs than high CFUs, whereas isolates with >75 CFUs will have more replicates with max CFU.

### Correlations between the evolution of tolerance and resistance

To determine if the evolution of resistance depends on tolerance, and/or vice versa, we use phytools in R with the fitPagel function, using the fitMk method, and an equal rates model (Revell, 2012). This function tests whether a model, where evolution of tolerance and resistance depends on each other, fits the data better than a model where the traits evolve independently, considering the phylogenetic distance between isolates. For each combination, we ran three models: whether there is an overall dependence between the traits, and whether one depends on the other or vice versa. We performed the analyses for DK1 and DK2 separately.

### Correlation of tolerance and resistance phenotypes between antibiotics

We also test whether there is a correlation between tolerance phenotypes between antibiotic treatments, e.g., is the evolution of tolerance to meropenem dependant on tolerance to ciprofloxacin? We do this with phytools in R, as described above.

We test if there is a difference in maximum OD, and sampling time, of resistant or tolerant isolates, and an interaction between the two factors, with a two-way ANOVA with Tukey post hoc comparisons.

### Statistical analysis

All statistical analyses and graphs were done in R version 3.3.1 (R Core Team, 2022), using packages broom (Robinson, 2021), gridextra (Auguie, 2015), ggbeeswarm (Eklund, 2021), and tidyverse (Wickham, 2019), including dplyr (Wickham et al., 2023a), ggplot2 (Wickham, 2016), readr (Wickham et al., 2023b), phytools (Revell, 2012), and forcats (Wickham et al., 2023c).

## Supplementary Material

qrad034_suppl_Supplementary_Material

qrad034_suppl_Supplementary_Tables

## Data Availability

All data are uploaded as supplementary materials.
