# Supplementary material for: Long-term evolution of antibiotic tolerance in Pseudomonas aeruginosa lung infections
Source: Evol Lett. 2023 Sep 20;7(6):389–400. doi: 10.1093/evlett/qrad034 (PMC10693005; doi:10.1093/evlett/qrad034)
Supplement: qrad034_suppl_Supplementary_Material [file qrad034_suppl_supplementary_material.pdf]

Supplementary table legends (tables in excel file)

Table S1: An overview of isolates of the transmissible clone types DK1 and DK2, with the sampling year, patient ID and time since first sampling of clone type. The tolerance and resistance data is divided by antibiotic, cipro = ciprofloxacin, mero = meropenem, tobra = tobramycin. For each isolate and drug is the overall type given (L = low tolerance, H = high tolerance, S = sensitive, R = resistant) and average CFU as undiluted (CFU1), diluted 10 fold (CFU10) and diluted 100 fold (CFU100), and average OD for each experiment, and the overall average OD per isolate. The MIC measured after 24h is given, and the resistance profile as sensitive, intermediate or resistant following the clinical cut-offs listed in the methods.

Table S2: Results from GLMs for antibiotic tolerance, testing the effect of time and max OD, and the interaction between the two. In blue is highlighted p-values < 0.05 and the best fit model with the lowest Akaike information criterion (AIC). Analysis for DK1 and DK2 separately.

Table S3: Results from GLMs for antibiotic resistance, testing the effect of time and max OD, and the interaction between the two. In blue is highlighted p-values < 0.05 and the best fit model with the lowest Akaike information criterion (AIC). Analysis for DK1 and DK2 separately.

Table S4: Results from 2-way ANOVA, testing the effect of max OD on tolerance and resistance, and the interaction between the two. Analysis for DK1 and DK2 separately.

Table S5: Results from 2-way ANOVA, testing the effect of length of infection on tolerance and resistance, and the interaction between the two. Analysis for DK1 and DK2 separately.

Supplementary figure legends

Fig. S1 A & B Phylogenies of DK1 and DK2, modified from Andersen et al. 2019 (DK1) and Marvig et al. 2013 (DK2). Each isolate is denoted by the sample year, patient ID and sample ID. The heat map shows the presence (red square) or absence (white square) of tolerance (T) and resistance (R) to the three antibiotics, in the order ciprofloxacin, meropenem, and tobramycin. Isolates with missing phenotypic data are shown with a strike-through. To ease interpretation, branch lengths are not drawn to scale. Isolates that only show high tolerance when the culture is diluted 10 times are shown in pink (only found in DK1).

Fig. S1A

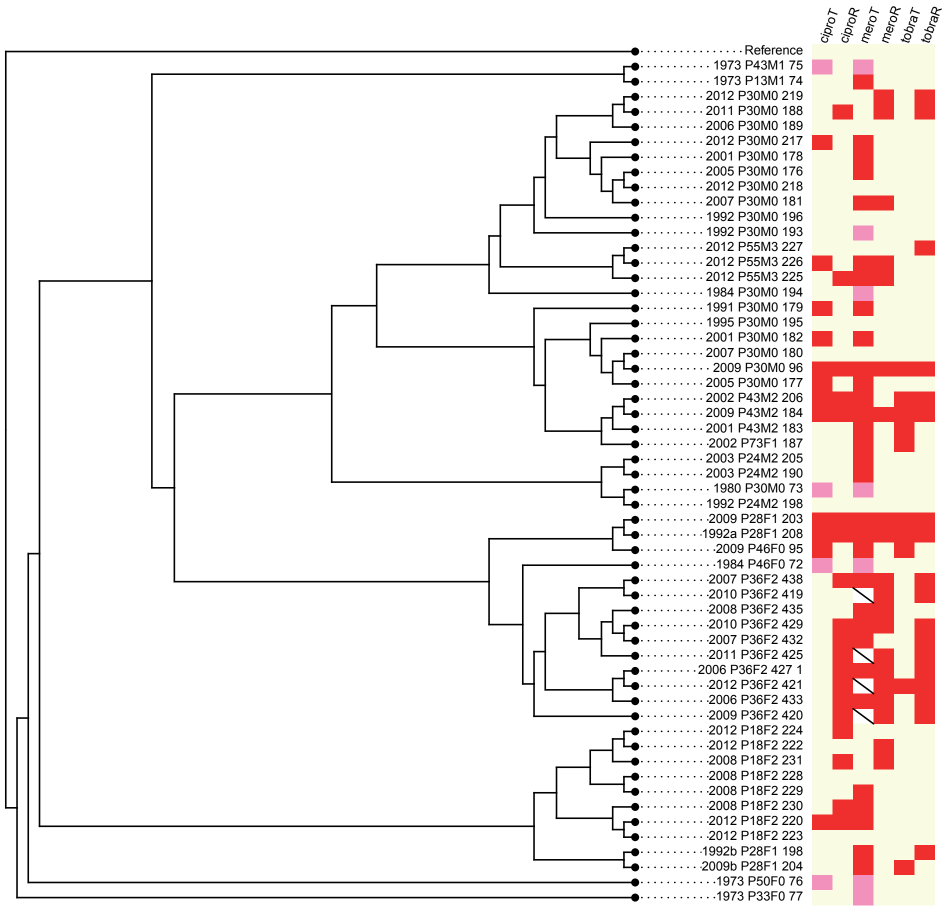

cipro*T*
cipro*R*
mero*T*
mero*R*
tobra*T*
tobra*R*
Reference
1973 P43M1 75
1973 P13M1 74
2012 P30M0 219
2011 P30M0 188
2006 P30M0 189
2012 P30M0 217
2001 P30M0 178
2005 P30M0 176
2012 P30M0 218
2007 P30M0 181
1992 P30M0 196
1992 P30M0 193
2012 P55M3 227
2012 P55M3 226
2012 P55M3 225
1984 P30M0 194
1991 P30M0 179
1995 P30M0 195
2001 P30M0 182
2007 P30M0 180
2009 P30M0 96
2005 P30M0 177
2002 P43M2 206
2009 P43M2 184
2001 P43M2 183
2002 P73F1 187
2003 P24M2 205
2003 P24M2 190
1980 P30M0 73
1992 P24M2 198
2009 P28F1 203
1992a P28F1 208
2009 P46F0 95
1984 P46F0 72
2007 P36F2 438
2010 P36F2 419
2008 P36F2 435
2010 P36F2 429
2007 P36F2 432
2011 P36F2 425
2006 P36F2 427 1
2012 P36F2 421
2006 P36F2 433
2009 P36F2 420
2012 P18F2 224
2012 P18F2 222
2008 P18F2 231
2008 P18F2 228
2008 P18F2 229
2008 P18F2 230
2012 P18F2 220
2012 P18F2 223
1992b P28F1 198
2009b P28F1 204
1973 P50F0 76
1973 P33F0 77

Fig. S1B

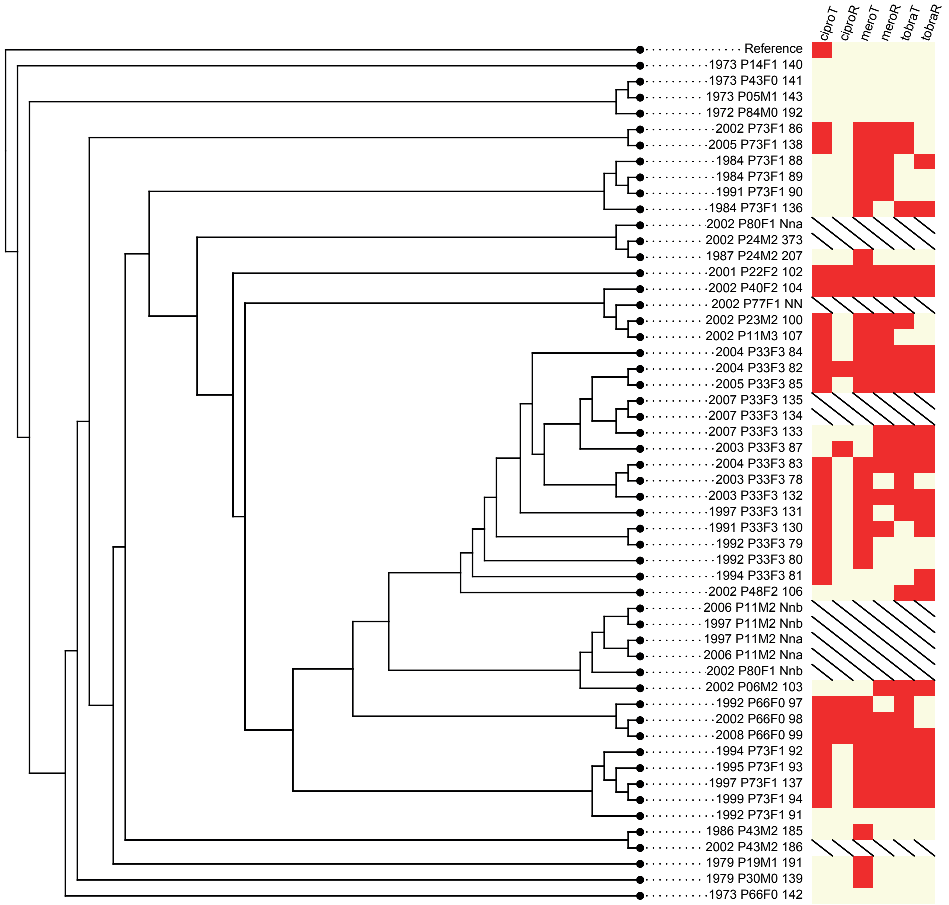

ciproT
ciproR
meroT
meroR
tobraT
tobraR
Reference
1973 P14F1 140
1973 P43F0 141
1973 P05M1 143
1972 P84M0 192
2002 P73F1 86
2005 P73F1 138
1984 P73F1 88
1984 P73F1 89
1991 P73F1 90
1984 P73F1 136
2002 P80F1 Nna
2002 P24M2 373
1987 P24M2 207
2001 P22F2 102
2002 P40F2 104
2002 P77F1 NN
2002 P23M2 100
2002 P11M3 107
2004 P33F3 84
2004 P33F3 82
2005 P33F3 85
2007 P33F3 135
2007 P33F3 134
2007 P33F3 133
2003 P33F3 87
2004 P33F3 83
2003 P33F3 78
2003 P33F3 132
1997 P33F3 131
1991 P33F3 130
1992 P33F3 79
1992 P33F3 80
1994 P33F3 81
2002 P48F2 106
2006 P11M2 Nnb
1997 P11M2 Nnb
1997 P11M2 Nna
2006 P11M2 Nna
2002 P80F1 Nnb
2002 P06M2 103
1992 P66F0 97
2002 P66F0 98
2008 P66F0 99
1994 P73F1 92
1995 P73F1 93
1997 P73F1 137
1999 P73F1 94
1992 P73F1 91
1986 P43M2 185
2002 P43M2 186
1979 P19M1 191
1979 P30M0 139
1973 P66F0 142

Fig. S2 Graphs show the difference in persistence measured as CFU counts from undiluted culture or culture diluted 10 times for isolates classified as low tolerance, high tolerance, and isolates that “revive” and become high tolerance only when antibiotic is diluted. Only the “revival” category has a mean difference above 50. Boxplots show median CFU counts ± 25 percentiles and values as dots.

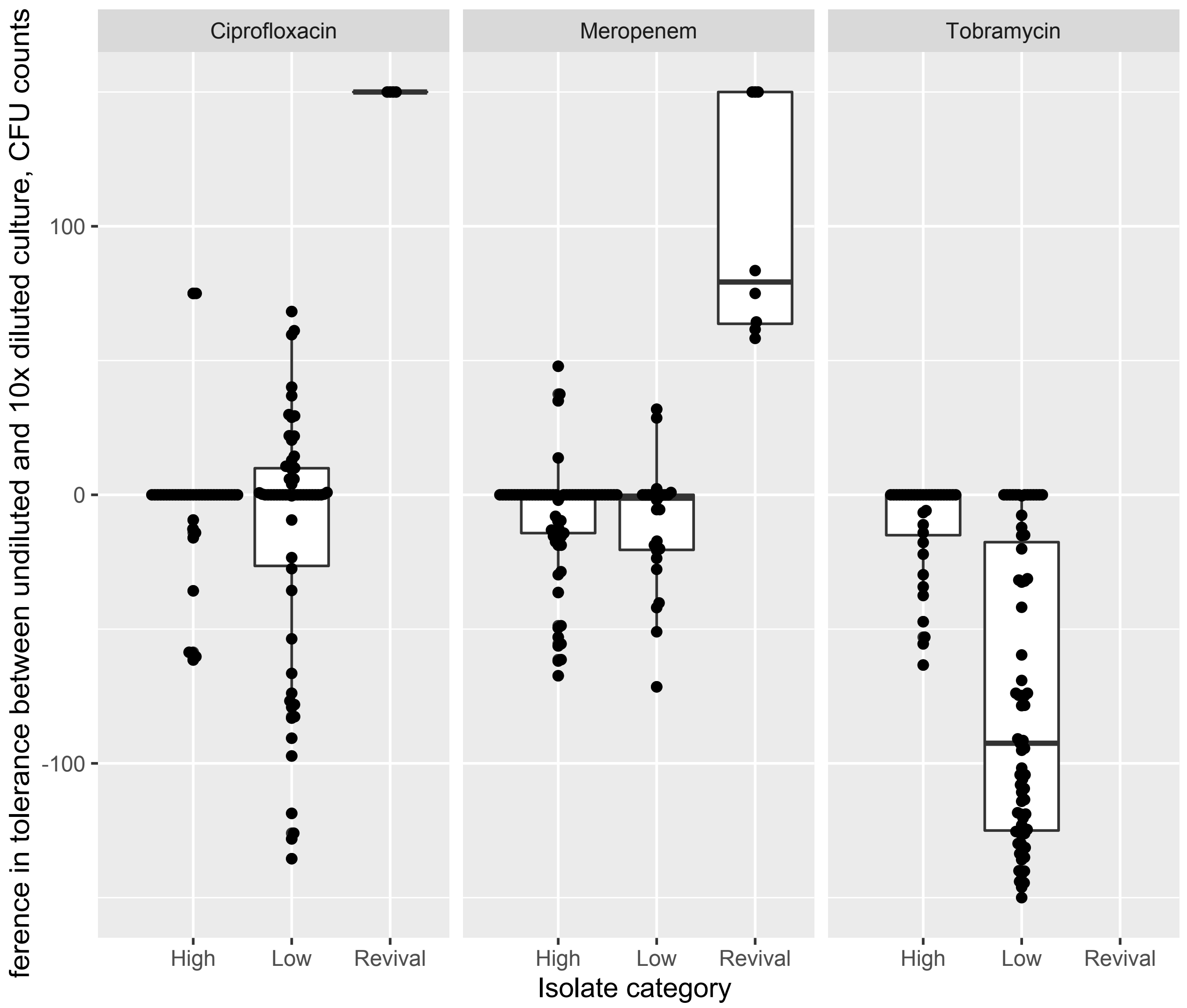

Fig. S3 Correlation between the mean CFU count at 10 times dilution, and the standard deviation around the mean. There is a bell-shaped association, as isolates with an intermediate count tend to represent isolates with replicates of either very high or very low counts. The dashed red line marks the cut-off of 75 CFU to classify isolates as either low or high tolerance.

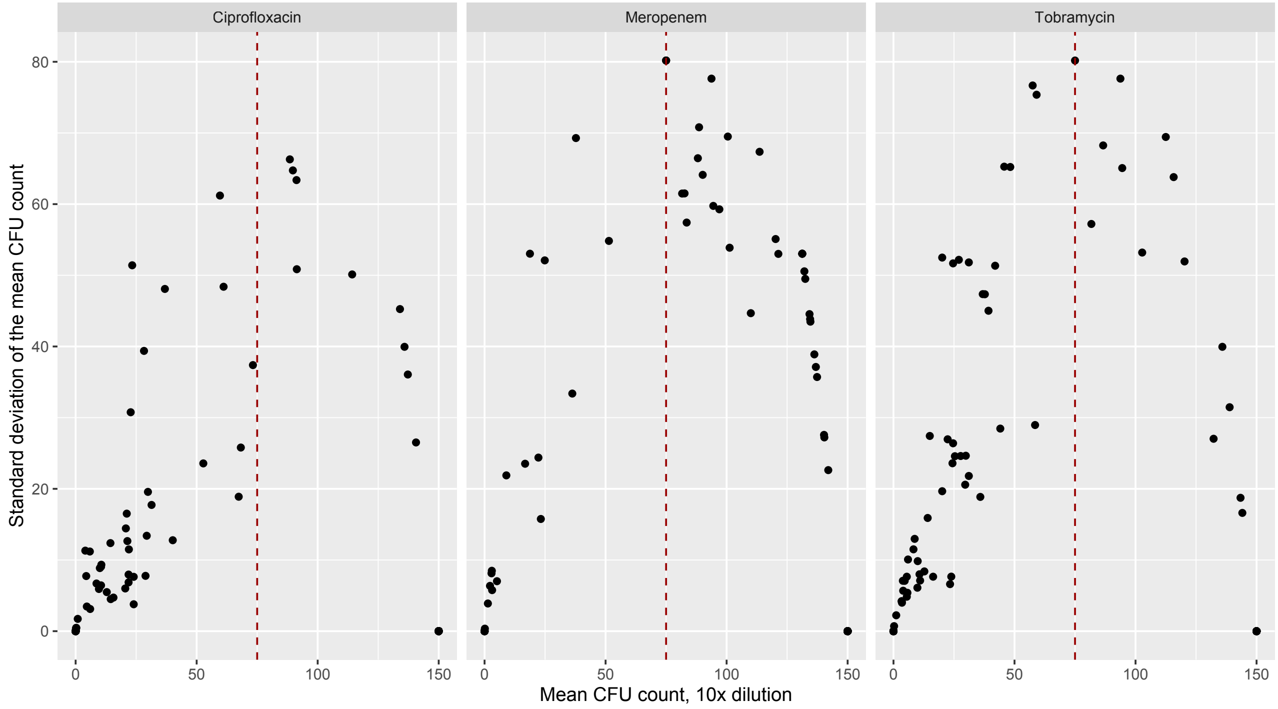

Supplementary text

We tested the correlation between the evolution of resistance and tolerance using the fitPagel function in phytools in R, using the fitMk method, and an equal rates model. We performed the analyses for DK1 and DK2 separately.

We found that for DK2, the evolution of resistance significantly depended on tolerance for ciprofloxacin and meropenem, as the transition from susceptible, nontolerant to resistant nontolerant, and vice versa was unlikely to occur. For DK1, tolerance to meropenem depended on significantly resistance, the transition from nontolerant resistant to tolerant resistant, and vice versa was unlikely to occur.

Between antibiotics, tolerance and resistance to ciprofloxacin and meropenem, and meropenem and tobramycin was correlated for DK1. For both DK1 and DK2 resistance to ciprofloxacin and tobramycin was correlated.

DK1, tolerance & resistance within antibiotic

```
> fit.DK1_cipro
Pagel's binary character correlation test:

Assumes "ER" substitution model for both characters

Independent model rate matrix:
                 ciproTol|ciproRes ciproTol|ciproSus ciproNotol|ciproRes ciproNotol|ciproSus
ciproTol|ciproRes           -44.7972           22.3986             22.3986              0.0000
ciproTol|ciproSus            22.3986          -44.7972              0.0000             22.3986
ciproNotol|ciproRes          22.3986            0.0000            -44.7972             22.3986
ciproNotol|ciproSus           0.0000           22.3986             22.3986            -44.7972

Dependent (x & y) model rate matrix:
                 ciproTol|ciproRes ciproTol|ciproSus ciproNotol|ciproRes ciproNotol|ciproSus
ciproTol|ciproRes         -26.919711           22.3986            4.521111              0.0000
ciproTol|ciproSus          22.398600          -44.7972            0.000000             22.3986
ciproNotol|ciproRes         4.521111            0.0000          -26.919711             22.3986
```

ciproNotol|ciproSus	0.000000	22.3986	22.398600	-44.7972

Model fit:

	log-likelihood	AIC
independent	-118.3225	240.6450
dependent	-117.2639	242.5278

Hypothesis test result:
  likelihood-ratio: 2.11713
  p-value: 0.346953

Model fitting method used was fitMk

> fit.DK1_cipro_x

Pagel's binary character correlation test:

Assumes "ER" substitution model for both characters

Independent model rate matrix:

	ciproTol|ciproRes	ciproTol|ciproSus	ciproNotol|ciproRes	ciproNotol|ciproSus
ciproTol|ciproRes	-44.7972	22.3986	22.3986	0.0000
ciproTol|ciproSus	22.3986	-44.7972	0.0000	22.3986
ciproNotol|ciproRes	22.3986	0.0000	-44.7972	22.3986
ciproNotol|ciproSus	0.0000	22.3986	22.3986	-44.7972

Dependent (x only) model rate matrix:

	ciproTol|ciproRes	ciproTol|ciproSus	ciproNotol|ciproRes	ciproNotol|ciproSus
ciproTol|ciproRes	-26.919591	22.3986	4.520991	0.0000
ciproTol|ciproSus	22.398600	-44.7972	0.000000	22.3986
ciproNotol|ciproRes	4.520991	0.0000	-26.919591	22.3986
ciproNotol|ciproSus	0.000000	22.3986	22.398600	-44.7972

Model fit:

	log-likelihood	AIC
independent	-118.3225	240.6450
dependent	-117.2639	240.5278

Hypothesis test result:
  likelihood-ratio: 2.11713
  p-value: 0.145659

Model fitting method used was fitMk

> fit.DK1_cipro_y

Pagel's binary character correlation test:

```
Assumes "ER" substitution model for both characters

Independent model rate matrix:
                    cipro Tol|ciproRes cipro Tol|ciproSus ciproNotol|ciproRes ciproNotol|ciproSus
cipro Tol|ciproRes          -44.7972          22.3986           22.3986            0.0000
cipro Tol|ciproSus           22.3986         -44.7972            0.0000           22.3986
ciproNotol|ciproRes          22.3986           0.0000          -44.7972           22.3986
ciproNotol|ciproSus           0.0000          22.3986           22.3986          -44.7972

Dependent (y only) model rate matrix:
                    cipro Tol|ciproRes cipro Tol|ciproSus ciproNotol|ciproRes ciproNotol|ciproSus
cipro Tol|ciproRes          -44.7972          22.3986           22.3986            0.0000
cipro Tol|ciproSus           22.3986         -44.7972            0.0000           22.3986
ciproNotol|ciproRes          22.3986           0.0000          -44.7972           22.3986
ciproNotol|ciproSus           0.0000          22.3986           22.3986          -44.7972

Model fit:
            log-likelihood     AIC
independent      -118.3225 240.645
dependent        -118.3225 242.645

Hypothesis test result:
  likelihood-ratio:  0
  p-value:  1

Model fitting method used was fitMk

> fit.DK1_mero

Pagel's binary character correlation test:

Assumes "ER" substitution model for both characters

Independent model rate matrix:
                  mero Tol|meroRes mero Tol|meroSus meroNotol|meroRes meroNotol|meroSus
mero Tol|meroRes          -44.7972          22.3986           22.3986            0.0000
mero Tol|meroSus           22.3986         -44.7972            0.0000           22.3986
meroNotol|meroRes          22.3986           0.0000          -44.7972           22.3986
meroNotol|meroSus           0.0000          22.3986           22.3986          -44.7972

Dependent (x & y) model rate matrix:
                  mero Tol|meroRes mero Tol|meroSus meroNotol|meroRes meroNotol|meroSus
mero Tol|meroRes          -22.3986          22.3986            0.0000            0.0000
mero Tol|meroSus           22.3986         -44.7972            0.0000           22.3986
meroNotol|meroRes           0.0000           0.0000          -22.3986           22.3986
meroNotol|meroSus           0.0000          22.3986           22.3986          -44.7972

Model fit:
```

	log-likelihood	AIC
independent	-115.9321	235.8643
dependent	-113.6624	235.3248

Hypothesis test result:
likelihood-ratio: 4.53949
p-value: 0.103339

Model fitting method used was fitMk

> fit.DK1_mero_x

Pagel's binary character correlation test:

Assumes "ER" substitution model for both characters

Independent model rate matrix:

	meroTol|meroRes	meroTol|meroSus	meroNotol|meroRes	meroNotol|meroSus
meroTol|meroRes	-44.7972	22.3986	22.3986	0.0000
meroTol|meroSus	22.3986	-44.7972	0.0000	22.3986
meroNotol|meroRes	22.3986	0.0000	-44.7972	22.3986
meroNotol|meroSus	0.0000	22.3986	22.3986	-44.7972

Dependent (x only) model rate matrix:

	meroTol|meroRes	meroTol|meroSus	meroNotol|meroRes	meroNotol|meroSus
meroTol|meroRes	-22.3986	22.3986	0.0000	0.0000
meroTol|meroSus	22.3986	-44.7972	0.0000	22.3986
meroNotol|meroRes	0.0000	0.0000	-22.3986	22.3986
meroNotol|meroSus	0.0000	22.3986	22.3986	-44.7972

Model fit:

	log-likelihood	AIC
independent	-115.9321	235.8643
dependent	-113.6624	233.3248

Hypothesis test result:
likelihood-ratio: 4.53949
p-value: 0.0331215

Model fitting method used was fitMk

> fit.DK1_mero_y

Pagel's binary character correlation test:

Assumes "ER" substitution model for both characters

Independent model rate matrix:

```
                    meroTol|meroRes meroTol|meroSus meroNotol|meroRes meroNotol|meroSus
meroTol|meroRes            -44.7972         22.3986           22.3986            0.0000
meroTol|meroSus             22.3986        -44.7972            0.0000           22.3986
meroNotol|meroRes           22.3986          0.0000          -44.7972           22.3986
meroNotol|meroSus            0.0000         22.3986           22.3986          -44.7972
```

Dependent (y only) model rate matrix:

```
                    meroTol|meroRes meroTol|meroSus meroNotol|meroRes meroNotol|meroSus
meroTol|meroRes            -44.7972         22.3986           22.3986            0.0000
meroTol|meroSus             22.3986        -44.7972            0.0000           22.3986
meroNotol|meroRes           22.3986          0.0000          -44.7972           22.3986
meroNotol|meroSus            0.0000         22.3986           22.3986          -44.7972
```

Model fit:

```
            log-likelihood      AIC
independent      -115.9321 235.8643
dependent        -115.9321 237.8643
```

Hypothesis test result:
  likelihood-ratio: 0
  p-value: 1

Model fitting method used was fitMk

```
> fit.DK1_tobra
```

Pagel's binary character correlation test:

Assumes "ER" substitution model for both characters

Independent model rate matrix:

```
                      tobraTol|tobraRes tobraTol|tobraSus tobraNotol|tobraRes tobraNotol|tobraSus
tobraTol|tobraRes             -30.92601          17.23918            13.68682             0.00000
tobraTol|tobraSus              17.23918         -30.92601             0.00000            13.68682
tobraNotol|tobraRes            13.68682           0.00000           -30.92601            17.23918
tobraNotol|tobraSus             0.00000          13.68682            17.23918           -30.92601
```

Dependent (x & y) model rate matrix:

```
                      tobraTol|tobraRes tobraTol|tobraSus tobraNotol|tobraRes tobraNotol|tobraSus
tobraTol|tobraRes            -23.650057          22.39860            1.251457             0.00000
tobraTol|tobraSus             22.398600         -44.08045            0.000000            21.68185
tobraNotol|tobraRes            1.251457           0.00000          -21.519523            20.26807
tobraNotol|tobraSus            0.000000          21.68185           20.268066           -41.94991
```

Model fit:

```
            log-likelihood      AIC
independent      -61.86093 127.7219
dependent        -60.48619 128.9724
```

Hypothesis test result:
  likelihood-ratio: 2.74948
  p-value: 0.252906

Model fitting method used was fitMk

> fit.DK1_tobra_x

Pagel's binary character correlation test:

Assumes "ER" substitution model for both characters

Independent model rate matrix:

	tobraTol|tobraRes	tobraTol|tobraSus	tobraNotol|tobraRes	tobraNotol|tobraSus
tobraTol|tobraRes	-30.92601	17.23918	13.68682	0.00000
tobraTol|tobraSus	17.23918	-30.92601	0.00000	13.68682
tobraNotol|tobraRes	13.68682	0.00000	-30.92601	17.23918
tobraNotol|tobraSus	0.00000	13.68682	17.23918	-30.92601

Dependent (x only) model rate matrix:

	tobraTol|tobraRes	tobraTol|tobraSus	tobraNotol|tobraRes	tobraNotol|tobraSus
tobraTol|tobraRes	-30.34306	15.63335	14.70971	0.00000
tobraTol|tobraSus	15.63335	-30.94598	0.00000	15.31263
tobraNotol|tobraRes	14.70971	0.00000	-30.34306	15.63335
tobraNotol|tobraSus	0.00000	15.31263	15.63335	-30.94598

Model fit:

	log-likelihood	AIC
independent	-61.86093	127.7219
dependent	-62.04903	130.0981

Hypothesis test result:
  likelihood-ratio: -0.376208
  p-value: 1

Model fitting method used was fitMk

> fit.DK1_tobra_y

Pagel's binary character correlation test:

Assumes "ER" substitution model for both characters

Independent model rate matrix:

	tobraTol|tobraRes	tobraTol|tobraSus	tobraNotol|tobraRes	tobraNotol|tobraSus
tobraTol|tobraRes	-30.92601	17.23918	13.68682	0.00000
tobraTol|tobraSus	17.23918	-30.92601	0.00000	13.68682

tobraNotol|tobraRes	13.68682	0.00000	-30.92601	17.23918
tobraNotol|tobraSus	0.00000	13.68682	17.23918	-30.92601

Dependent (y only) model rate matrix:

	tobraTol|tobraRes	tobraTol|tobraSus	tobraNotol|tobraRes	tobraNotol|tobraSus
tobraTol|tobraRes	-30.83490	15.81245	15.02245	0.00000
tobraTol|tobraSus	15.81245	-30.83490	0.00000	15.02245
tobraNotol|tobraRes	15.02245	0.00000	-30.13893	15.11648
tobraNotol|tobraSus	0.00000	15.02245	15.11648	-30.13893

Model fit:

	log-likelihood	AIC
independent	-61.86093	127.7219
dependent	-62.11166	130.2233

Hypothesis test result:
 likelihood-ratio: -0.501458
 p-value: 1

Model fitting method used was fitMk

DK1, between antibiotics

> fit.DK1_merotobraRes
Pagel's binary character correlation test:

Assumes "ER" substitution model for both characters

Independent model rate matrix:

	meroRes|tobraRes	meroRes|tobraSus	meroSus|tobraRes	meroSus|tobraSus
meroRes|tobraRes	-31.23985	15.47715	15.76271	0.00000
meroRes|tobraSus	15.47715	-31.23985	0.00000	15.76271
meroSus|tobraRes	15.76271	0.00000	-31.23985	15.47715
meroSus|tobraSus	0.00000	15.76271	15.47715	-31.23985

Dependent (x & y) model rate matrix:

	meroRes|tobraRes	meroRes|tobraSus	meroSus|tobraRes	meroSus|tobraSus
meroRes|tobraRes	-26.5963	4.1977	22.3986	0.0000
meroRes|tobraSus	4.1977	-26.5963	0.0000	22.3986
meroSus|tobraRes	22.3986	0.0000	-44.7972	22.3986
meroSus|tobraSus	0.0000	22.3986	22.3986	-44.7972

Model fit:

	log-likelihood	AIC
independent	-79.19063	162.3813
dependent	-76.48482	160.9696

```
Hypothesis test result:
  likelihood-ratio:  5.41162
  p-value:  0.0668161

Model fitting method used was fitMk

> fit.DK1_merotobraRes_x

Pagel's binary character correlation test:

Assumes "ER" substitution model for both characters

Independent model rate matrix:
                  meroRes|tobraRes meroRes|tobraSus meroSus|tobraRes meroSus|tobraSus
meroRes|tobraRes        -31.23985         15.47715         15.76271          0.00000
meroRes|tobraSus         15.47715        -31.23985          0.00000         15.76271
meroSus|tobraRes         15.76271          0.00000        -31.23985         15.47715
meroSus|tobraSus          0.00000         15.76271         15.47715        -31.23985

Dependent (x only) model rate matrix:
                  meroRes|tobraRes meroRes|tobraSus meroSus|tobraRes meroSus|tobraSus
meroRes|tobraRes         -44.7972          22.3986          22.3986           0.0000
meroRes|tobraSus          22.3986         -44.7972           0.0000          22.3986
meroSus|tobraRes          22.3986           0.0000         -44.7972          22.3986
meroSus|tobraSus           0.0000          22.3986          22.3986         -44.7972

Model fit:
            log-likelihood      AIC
independent      -79.19063 162.3813
dependent        -76.97216 159.9443

Hypothesis test result:
  likelihood-ratio:  4.43695
  p-value:  0.0351689

Model fitting method used was fitMk

> fit.DK1_merotobraRes_y

Pagel's binary character correlation test:

Assumes "ER" substitution model for both characters

Independent model rate matrix:
                  meroRes|tobraRes meroRes|tobraSus meroSus|tobraRes meroSus|tobraSus
meroRes|tobraRes        -31.23985         15.47715         15.76271          0.00000
meroRes|tobraSus         15.47715        -31.23985          0.00000         15.76271
```

meroSus|tobraRes	15.76271	0.00000	-31.23985	15.47715
meroSus|tobraSus	0.00000	15.76271	15.47715	-31.23985

Dependent (y only) model rate matrix:

	meroRes|tobraRes	meroRes|tobraSus	meroSus|tobraRes	meroSus|tobraSus
meroRes|tobraRes	-32.73438	10.33999	22.39439	0.00000
meroRes|tobraSus	10.33999	-32.73438	0.00000	22.39439
meroSus|tobraRes	22.39439	0.00000	-38.43961	16.04522
meroSus|tobraSus	0.00000	22.39439	16.04522	-38.43961

Model fit:

	log-likelihood	AIC
independent	-79.19063	162.3813
dependent	-77.31781	160.6356

Hypothesis test result:
 likelihood-ratio: 3.74565
 p-value: 0.052945

Model fitting method used was fitMk

> fit.DK1_cipromeroTol

Pagel's binary character correlation test:

Assumes "ER" substitution model for both characters

Independent model rate matrix:

	ciproTol|meroTol	ciproTol|meroNotol	ciproNotol|meroTol
ciproTol|meroTol	-29.50124	14.75062	14.75062
ciproTol|meroNotol	14.75062	-29.50124	0.00000
ciproNotol|meroTol	14.75062	0.00000	-29.50124
ciproNotol|meroNotol	0.00000	14.75062	14.75062

	ciproNotol|meroNotol
ciproTol|meroTol	0.00000
ciproTol|meroNotol	14.75062
ciproNotol|meroTol	14.75062
ciproNotol|meroNotol	-29.50124

Dependent (x & y) model rate matrix:

	ciproTol|meroTol	ciproTol|meroNotol	ciproNotol|meroTol
ciproTol|meroTol	-22.3986	0	22.3986
ciproTol|meroNotol	0.0000	0	0.0000
ciproNotol|meroTol	22.3986	0	-44.7972
ciproNotol|meroNotol	0.0000	0	22.3986

	ciproNotol|meroNotol
ciproTol|meroTol	0.0000
ciproTol|meroNotol	0.0000

```
cipro Notol|meroTol              22.3986
cipro Notol|meroNotol           -22.3986

Model fit:
           log-likelihood      AIC
independent     -149.2676 302.5352
dependent       -140.0877 288.1753

Hypothesis test result:
  likelihood-ratio:  18.3599
  p-value:  0.000103086

Model fitting method used was fitMk

> fit.DK1_cipromeroTol_x

Pagel's binary character correlation test:

Assumes "ER" substitution model for both characters

Independent model rate matrix:
                  ciproTol|meroTol ciproTol|meroNotol ciproNotol|meroTol
ciproTol|meroTol          -29.50124           14.75062           14.75062
ciproTol|meroNotol         14.75062          -29.50124            0.00000
ciproNotol|meroTol         14.75062            0.00000          -29.50124
ciproNotol|meroNotol        0.00000           14.75062           14.75062
                  ciproNotol|meroNotol
ciproTol|meroTol               0.00000
ciproTol|meroNotol            14.75062
ciproNotol|meroTol            14.75062
ciproNotol|meroNotol         -29.50124

Dependent (x only) model rate matrix:
                  ciproTol|meroTol ciproTol|meroNotol ciproNotol|meroTol
ciproTol|meroTol           -44.7972           22.39860           22.3986
ciproTol|meroNotol          22.3986          -37.40151            0.0000
ciproNotol|meroTol          22.3986            0.00000          -44.7972
ciproNotol|meroNotol         0.0000           15.00291           22.3986
                  ciproNotol|meroNotol
ciproTol|meroTol               0.00000
ciproTol|meroNotol            15.00291
ciproNotol|meroTol            22.39860
ciproNotol|meroNotol         -37.40151

Model fit:
           log-likelihood      AIC
independent     -149.2676 302.5352
dependent       -143.0984 292.1967
```

```
Hypothesis test result:
  likelihood-ratio:  12.3385
  p-value:  0.000443709

Model fitting method used was fitMk

> fit.DK1_cipromeroTol_y

Pagel's binary character correlation test:

Assumes "ER" substitution model for both characters

Independent model rate matrix:
                    ciproTol|meroTol ciproTol|meroNotol ciproNotol|meroTol
ciproTol|meroTol           -29.50124           14.75062           14.75062
ciproTol|meroNotol          14.75062          -29.50124            0.00000
ciproNotol|meroTol          14.75062            0.00000          -29.50124
ciproNotol|meroNotol         0.00000           14.75062           14.75062
                    ciproNotol|meroNotol
ciproTol|meroTol                 0.00000
ciproTol|meroNotol              14.75062
ciproNotol|meroTol              14.75062
ciproNotol|meroNotol           -29.50124

Dependent (y only) model rate matrix:
                    ciproTol|meroTol ciproTol|meroNotol ciproNotol|meroTol
ciproTol|meroTol           -29.50124           14.75062           14.75062
ciproTol|meroNotol          14.75062          -29.50124            0.00000
ciproNotol|meroTol          14.75062            0.00000          -29.50124
ciproNotol|meroNotol         0.00000           14.75062           14.75062
                    ciproNotol|meroNotol
ciproTol|meroTol                 0.00000
ciproTol|meroNotol              14.75062
ciproNotol|meroTol              14.75062
ciproNotol|meroNotol           -29.50124

Model fit:
            log-likelihood      AIC
independent      -149.2676 302.5352
dependent        -149.2676 304.5352

Hypothesis test result:
  likelihood-ratio:  4.82917e-07
  p-value:  0.999446

Model fitting method used was fitMk
```

```
> fit.DK1_ciprotobraTol

Pagel's binary character correlation test:

Assumes "ER" substitution model for both characters

Independent model rate matrix:
                   ciproTol|tobraTol ciproTol|tobraNotol ciproNotol|tobraTol
ciproTol|tobraTol          -33.00845           10.60985           22.39860
ciproTol|tobraNotol         10.60985          -33.00845            0.00000
ciproNotol|tobraTol         22.39860            0.00000          -33.00845
ciproNotol|tobraNotol        0.00000           22.39860           10.60985
                   ciproNotol|tobraNotol
ciproTol|tobraTol                0.00000
ciproTol|tobraNotol             22.39860
ciproNotol|tobraTol             10.60985
ciproNotol|tobraNotol          -33.00845

Dependent (x & y) model rate matrix:
                   ciproTol|tobraTol ciproTol|tobraNotol ciproNotol|tobraTol
ciproTol|tobraTol           -44.7972            22.3986           22.398600
ciproTol|tobraNotol          22.3986           -44.7972            0.000000
ciproNotol|tobraTol          22.3986             0.0000          -29.204605
ciproNotol|tobraNotol         0.0000            22.3986            6.806005
                   ciproNotol|tobraNotol
ciproTol|tobraTol               0.000000
ciproTol|tobraNotol            22.398600
ciproNotol|tobraTol             6.806005
ciproNotol|tobraNotol         -29.204605

Model fit:
            log-likelihood      AIC
independent      -95.85530 195.7106
dependent        -95.32478 198.6496

Hypothesis test result:
  likelihood-ratio:  1.06105
  p-value:  0.588297

Model fitting method used was fitMk

> fit.DK1_ciprotobraTol_x

Pagel's binary character correlation test:

Assumes "ER" substitution model for both characters

Independent model rate matrix:
```

```
                  ciproTol|tobraTol ciproTol|tobraNotol ciproNotol|tobraTol
ciproTol|tobraTol           -33.00845           10.60985           22.39860
ciproTol|tobraNotol          10.60985          -33.00845            0.00000
ciproNotol|tobraTol          22.39860            0.00000          -33.00845
ciproNotol|tobraNotol         0.00000           22.39860           10.60985
                  ciproNotol|tobraNotol
ciproTol|tobraTol                0.00000
ciproTol|tobraNotol             22.39860
ciproNotol|tobraTol             10.60985
ciproNotol|tobraNotol          -33.00845

Dependent (x only) model rate matrix:
                  ciproTol|tobraTol ciproTol|tobraNotol ciproNotol|tobraTol
ciproTol|tobraTol           -18.58443           10.77089            7.81354
ciproTol|tobraNotol          10.77089          -33.16949            0.00000
ciproNotol|tobraTol           7.81354            0.00000          -18.58443
ciproNotol|tobraNotol         0.00000           22.39860           10.77089
                  ciproNotol|tobraNotol
ciproTol|tobraTol                0.00000
ciproTol|tobraNotol             22.39860
ciproNotol|tobraTol             10.77089
ciproNotol|tobraNotol          -33.16949

Model fit:
            log-likelihood      AIC
independent      -95.85530 195.7106
dependent        -95.76702 197.5340

Hypothesis test result:
  likelihood-ratio:  0.176556
  p-value:  0.67435

Model fitting method used was fitMk

> fit.DK1_ciprotobraTol_y

Pagel's binary character correlation test:

Assumes "ER" substitution model for both characters

Independent model rate matrix:
                  ciproTol|tobraTol ciproTol|tobraNotol ciproNotol|tobraTol
ciproTol|tobraTol           -33.00845           10.60985           22.39860
ciproTol|tobraNotol          10.60985          -33.00845            0.00000
ciproNotol|tobraTol          22.39860            0.00000          -33.00845
ciproNotol|tobraNotol         0.00000           22.39860           10.60985
                  ciproNotol|tobraNotol
ciproTol|tobraTol                0.00000
```

```
ciproTol|tobraNotol            22.39860
ciproNotol|tobraTol             10.60985
ciproNotol|tobraNotol          -33.00845

Dependent (y only) model rate matrix:
                   ciproTol|tobraTol ciproTol|tobraNotol ciproNotol|tobraTol
ciproTol|tobraTol             -44.7972            22.3986            22.398600
ciproTol|tobraNotol            22.3986           -44.7972             0.000000
ciproNotol|tobraTol            22.3986             0.0000           -29.204616
ciproNotol|tobraNotol           0.0000            22.3986             6.806016
                   ciproNotol|tobraNotol
ciproTol|tobraTol               0.000000
ciproTol|tobraNotol            22.398600
ciproNotol|tobraTol             6.806016
ciproNotol|tobraNotol         -29.204616

Model fit:
            log-likelihood      AIC
independent      -95.85530 195.7106
dependent        -95.32478 196.6496

Hypothesis test result:
  likelihood-ratio:  1.06105
  p-value:  0.302977

Model fitting method used was fitMk

> fit.DK1_merotobraTol

Pagel's binary character correlation test:

Assumes "ER" substitution model for both characters

Independent model rate matrix:
                  meroTol|tobraTol meroTol|tobraNotol meroNotol|tobraTol
meroTol|tobraTol          -33.00846           10.60986           22.39860
meroTol|tobraNotol         10.60986          -33.00846            0.00000
meroNotol|tobraTol         22.39860            0.00000          -33.00846
meroNotol|tobraNotol        0.00000           22.39860           10.60986
                  meroNotol|tobraNotol
meroTol|tobraTol                0.00000
meroTol|tobraNotol             22.39860
meroNotol|tobraTol             10.60986
meroNotol|tobraNotol          -33.00846

Dependent (x & y) model rate matrix:
                  meroTol|tobraTol meroTol|tobraNotol meroNotol|tobraTol
meroTol|tobraTol          -13.43302           13.43302                  0
```

```
meroTol|tobraNotol        13.43302        -35.83162              0
meroNotol|tobraTol         0.00000         0.00000             0
meroNotol|tobraNotol        0.00000         22.39860              0
                    meroNotol|tobraNotol
meroTol|tobraTol                0.0000
meroTol|tobraNotol              22.3986
meroNotol|tobraTol               0.0000
meroNotol|tobraNotol            -22.3986

Model fit:
            log-likelihood      AIC
independent     -100.42049 204.8410
dependent       -96.52869 201.0574

Hypothesis test result:
  likelihood-ratio:  7.78359
  p-value:  0.0204086

Model fitting method used was fitMk

> fit.DK1_merotobraTol_x

Pagel's binary character correlation test:

Assumes "ER" substitution model for both characters

Independent model rate matrix:
                    meroTol|tobraTol meroTol|tobraNotol meroNotol|tobraTol
meroTol|tobraTol          -33.00846         10.60986         22.39860
meroTol|tobraNotol         10.60986         -33.00846          0.00000
meroNotol|tobraTol         22.39860           0.00000        -33.00846
meroNotol|tobraNotol        0.00000          22.39860          10.60986
                    meroNotol|tobraNotol
meroTol|tobraTol               0.00000
meroTol|tobraNotol             22.39860
meroNotol|tobraTol             10.60986
meroNotol|tobraNotol           -33.00846

Dependent (x only) model rate matrix:
                    meroTol|tobraTol meroTol|tobraNotol meroNotol|tobraTol
meroTol|tobraTol          -8.559625          8.559625          0.000000
meroTol|tobraNotol         8.559625        -30.958225           0.000000
meroNotol|tobraTol         0.000000          0.000000          -8.559625
meroNotol|tobraNotol        0.000000          22.398600           8.559625
                    meroNotol|tobraNotol
meroTol|tobraTol              0.000000
meroTol|tobraNotol            22.398600
meroNotol|tobraTol             8.559625
```

```
meroNotol|tobraNotol         -30.958225

Model fit:
           log-likelihood      AIC
independent     -100.4205 204.8410
dependent       -97.5421 201.0842

Hypothesis test result:
  likelihood-ratio:  5.75678
  p-value:  0.0164251

Model fitting method used was fitMk

> fit.DK1_merotobraTol_y

Pagel's binary character correlation test:

Assumes "ER" substitution model for both characters

Independent model rate matrix:
                   meroTol|tobraTol meroTol|tobraNotol meroNotol|tobraTol
meroTol|tobraTol          -33.00846          10.60986          22.39860
meroTol|tobraNotol          10.60986          -33.00846            0.00000
meroNotol|tobraTol          22.39860            0.00000          -33.00846
meroNotol|tobraNotol          0.00000          22.39860            10.60986
                   meroNotol|tobraNotol
meroTol|tobraTol                  0.00000
meroTol|tobraNotol                22.39860
meroNotol|tobraTol                10.60986
meroNotol|tobraNotol             -33.00846

Dependent (y only) model rate matrix:
                   meroTol|tobraTol meroTol|tobraNotol meroNotol|tobraTol
meroTol|tobraTol          -38.47706          16.07846           22.3986
meroTol|tobraNotol          16.07846          -38.47706             0.0000
meroNotol|tobraTol          22.39860            0.00000           -22.3986
meroNotol|tobraNotol          0.00000          22.39860             0.0000
                   meroNotol|tobraNotol
meroTol|tobraTol                   0.0000
meroTol|tobraNotol                 22.3986
meroNotol|tobraTol                  0.0000
meroNotol|tobraNotol              -22.3986

Model fit:
           log-likelihood     AIC
independent    -100.42049 204.841
dependent       -99.76952 205.539
```

Hypothesis test result:
  likelihood-ratio: 1.30194
  p-value: 0.253859

Model fitting method used was fitMk
